# Effect of the Brugada syndrome mutation A39V on calmodulin regulation of Cav1.2 channels

**DOI:** 10.1186/1756-6606-7-34

**Published:** 2014-04-28

**Authors:** Brett A Simms, Ivana Assis Souza, Gerald W Zamponi

**Affiliations:** 1 Department of Physiology and Pharmacology, Hotchkiss Brain Institute, University of Calgary, 3330 Hospital Dr. NW, Calgary T2N 4N1, Canada

**Keywords:** Calcium channel, Calmodulin mutant, CDI, N-terminus, Brugada, Activation, Cav1.2, L-type, IQ, Channelopathy, Voltage, Gating, CACNA1C

## Abstract

**Background:**

The L-type calcium channel Cav1.2 is important for brain and heart function. The ubiquitous calcium sensing protein calmodulin (CaM) regulates calcium dependent gating of Cav1.2 channels by reducing calcium influx, a process known as calcium-dependent inactivation (CDI). Dissecting the calcium-dependence of CaM in this process has benefited greatly from the use of mutant CaM molecules which are unable to bind calcium to their low affinity (N-lobe) and high affinity (C-lobe) binding sites. Unlike CDI, it is unknown whether CaM can modulate the activation gating of Cav1.2 channels.

**Results:**

We examined a Cav1.2 point mutant in the N-terminus region of the channel (A39V) that has been previously linked to Brugada syndrome. Using mutant CaM constructs in which the N- and/or C-lobe calcium binding sites were ablated, we were able to show that this Brugada syndrome mutation disrupts N-lobe CDI of the channel. In the course of these experiments, we discovered that all mutant CaM molecules were able to alter the kinetics of channel activation even in the absence of calcium for WT-Cav1.2, but not A39V-Cav1.2 channels. Moreover, CaM mutants differentially shifted the voltage-dependence of activation for WT and A39V-Cav1.2 channels to hyperpolarized potentials. Our data therefore suggest that structural changes in CaM that arise directly from site directed mutagenesis of calcium binding domains alter activation gating of Cav1.2 channels independently of their effects on calcium binding, and that the N-terminus of the channel contributes to this CaM dependent process.

**Conclusions:**

Our data indicate that caution must be exercised when interpreting the effects of CaM mutants on ion channel gating.

## Background

Voltage-gated calcium channels (VGCCs) are important for modulating excitability, development and gene transcription in neurons [1] while dysfunction of these channels results in a host of neurological illnesses [2]. Conditional knockout of *CACNA1C* from murine cortex demonstrates that Cav1.2 has a central role in emotional learning, specifically fear conditioning and empathy [3,4]. In the heart Cav1.2 channels are essential for cardiac contraction [5-7], which is best demonstrated by its embryonic lethal knockout [8]. Also, many mutations in *CACNA1C* have been linked to Brugada syndrome, a cardiac disorder that is characterized by ventricular arrhythmia [9-11].

Altered trafficking of VGCCs to the cell membrane or aberrant function once at the cell surface are the most common molecular deficits underling disease [12]. Too little calcium conductance reduces neuronal excitability and gene transcription, while too much calcium entry is cytotoxic [13]. Excessive calcium influx through wild type Cav1.2 (WT-Cav1.2) channels is limited by the ubiquitous calcium sensing protein calmodulin (CaM), which promotes calcium-dependent inactivation (CDI) [14-16]. Deciphering calcium/calmodulin (Ca^2+^/CaM) dependent gating of various ion channels [17-20], including the complexities of Cav1.2 CDI [21-25] and trafficking [26], has benefited greatly from the use of CaM molecules with mutated low-affinity (N-lobe), or high affinity (C-lobe) calcium binding sites. Each lobe of CaM has two EF-hand motifs which when mutated (CaM_12_ is the N-lobe mutant and CaM_34_ is the C-lobe mutant) prevent the binding of calcium. CaM mutants unable to bind calcium have different structural properties [27,28] from those of wild type CaM molecules [29-32], suggesting that these conformational changes might affect channel gating independently of their ability, or inability to bind calcium.

Years of work with CaM mutants has shown that L-type calcium channels have multiple N-terminal [22,23,33] and C-terminal [24,34-37] CaM binding sites which functionally regulate global and local CDI, respectively. While investigating effects on global CDI for a Cav1.2 N-terminal point mutant (A39V) linked to Brugada syndrome [9,38] we observed that CaM_WT_ differentially affected the kinetics and voltage-dependence of activation for Cav1.2 when compared to CaM lobe mutants. We also show that these effects occur in the absence of calcium and that they can be modulated by the N-terminal A39V mutation, indicating that the N-terminus of the channel can be involved in CaM-dependent modulation of Cav1.2 activation.

## Results

### The Brugada syndrome mutant A39V disrupts N-lobe CDI of Cav1.2 channels but not CaM binding to the channel N-terminus

We have shown in previous work that the Cav1.2 point mutant A39V linked to Brugada syndrome, does not elicit a trafficking defect in the neuronal isoform of the channel or major effects on voltage-dependent activation and inactivation [38]. Recently Dick and colleagues [22] and our group [23] have shown that the N-terminus of L-type calcium channels participates in a type of CDI which occurs when intracellular levels of calcium elevate globally – it is therefore termed global CDI. Global CDI of Cav1.2 channels relies on the N-lobe of CaM, which has a much lower affinity for calcium than the high affinity C-lobe. In order to study global CDI of Cav1.2 channels the C-lobe of CaM must be rendered non-functional (i.e. CaM_34_) and intracellular calcium buffering made permissive for the N-lobe of CaM to bind calcium (0.5 EGTA). We coexpressed Cavβ2a and Cavα2δ1 subunits in our structure/function analysis of A39V-Cav1.2 because this combination of auxiliary subunits is regularly used to isolate CDI [15,22,23,39,40] due to the fact that Cavβ2a slows VDI [41-43] unlike Cavβ1b [14].

**Figure 1:**
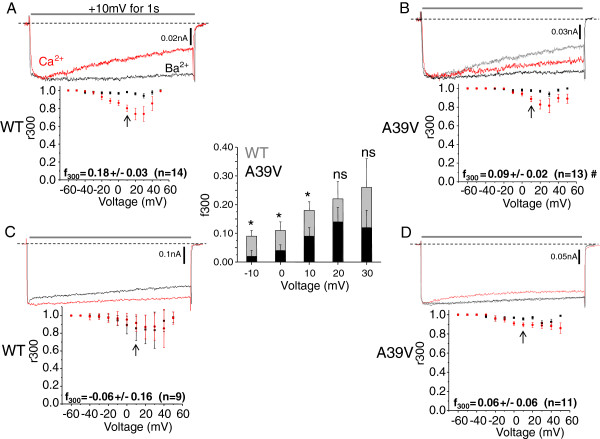
**The Brugada syndrome mutation A39V disrupts N-lobe CDI of Cav1.2 channels. A)** Representative Ba^2+^ (black) and Ca^2+^ (red) traces of WT and A39V-Cav1.2 channels **(B)** expressed with Cavβ2a/Cavα2δ in the presence of low calcium buffering (0.5 mM EGTA) and CaM_34_. Note that the peak of the Ba^2+^ trace is normalized to that in the presence of Ca^2+^. For reference the WT-Cav1.2 Ca^2+^ trace is displayed in grey in **(B)**. The plots shown below the current traces reflect average CDI (f_300_) which is quantified by the fraction of current remaining after 300 ms (r300) in calcium, and is then subtracted from the fraction of current remaining in barium at the same time point. The f_300_ value at 10 mV (arrows) is significantly less for A39V, than WT-Cav1.2 (# p ≤ 0.04 by student’s *t*-test). The inset bar graph displays additional f_300_ values over a potential range from -10 mV to +30 mV. A significant difference is observed in the f_300_ values between WT-Cav1.2 and A39V-Cav1.2 at −10, 0 and +10 mV (p ≤ 0.05 by student’s *t*-test), but not at +20 (p = 0.28 by student’s *t*-test) or +30 mV (p = 0.26 by student’s *t*-test). **C)** Under high calcium buffering (10 mM BAPTA) conditions WT-Cav1.2 channels expressed as in **(A)** no longer exhibit N-lobe CDI. **D)** A39V-Cav1.2 does not show significant N-lobe CDI compared to WT-Cav1.2 in high calcium buffering at +10 mV (arrows). For reference the WT-Cav1.2 Ca^2+^ trace **(C)** is displayed in grey.

Because of the documented role of the Cav1.2 N-terminus in CDI, we tested whether the Brugada syndrome mutant A39Vmay affect this process. This was done by overexpressing CaM_34_ and buffering intracellular calcium with 0.5 mM EGTA. Figure 1A shows that WT-Cav1.2 channels show little voltage-dependent inactivation (VDI) in barium (black trace) but significant N-lobe CDI upon exposure to calcium (red trace), in agreement with our previous work [23]. A +10 mV test depolarization was used for comparison in Figure 1 because this test potential corresponds to the peak of the IV curve in 20 mM external barium. Quantification of the amount of N-lobe CDI is reflected in the f_300_ value (i.e. the fraction of channels which are inactivated after 300 ms) at +10 mV, which equals 0.18 ± 0.03 (n = 14) for WT-Cav1.2 and CaM_34_. Figure 1B shows that A39V-Cav1.2 channels have significantly reduced N-lobe CDI at +10 mV (f_300_ = 0.09 ± 0.02, n = 13, # p ≤0.04 by student’s *t*-test) with CaM_34_ compared to WT-Cav1.2 channels under the same conditions. To facilitate comparison the WT-Cav1.2 calcium trace is shown in grey in Figure 1B. The inset bar graph in Figure 1 shows that in addition to +10 mV, A39V-Cav1.2 shows significantly less CDI than WT-Cav1.2 at −10 and 0 mV (p ≤ 0.05 by student’s *t*-test). A similar trend was seen +20 and +30 mV, but did not reach statistical significance (p = 0.28 and p = 0.26 by student’s *t*-test, respectively), presumably because there is an increasing contribution of VDI at these potentials. Repeating the experiment with 10 mM BAPTA intracellularly to significantly increase calcium buffering verified that both WT-Cav1.2 (f_300_ = −0.06 ± 0.16, n = 9) (Figure 1C) and A39V-Cav1.2 (f_300_ = 0.06 ± 0.06, n = 11) (Figure 1D) channels were indeed undergoing N-lobe CDI at +10 mV, which is also supported by the flattened red traces in Figures 1C/D.

**Figure 2:**
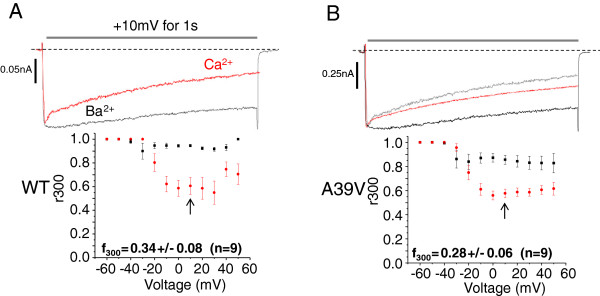
**The Brugada syndrome mutation A39V does not affect C-lobe CDI of Cav1.2 channels. A)** Representative Ba^2+^ (black) and Ca^2+^ (red) traces of WT-Cav1.2 channels expressed with Cavβ2a/Cavα2δ and CaM_12_ in the presence of high calcium buffering (10 mM BAPTA). Note that the peak of the Ba^2+^ trace is normalized to that in the presence of Ca^2+^ and that average CDI (f_300_) of WT-Cav1.2 is displayed below in the graph. **B)** A39V-Cav1.2 channels expressed with Cavβ2a/Cavα2δ and CaM_12_ in the presence of high calcium buffering (10 mM BAPTA). Inset graphs show average CDI (f_300_) which is not statistically different (p ≤ 0.55 by student’s *t*-test) between the two channel types.

We next tested whether A39V-Cav1.2 channels exhibited augmented C-lobe CDI by expressing the channels with CaM_12_. Figure 2A shows that WT-Cav1.2 channels show substantial C-lobe CDI in the presence of calcium (f_300_ = 0.34 ± 0.08, n = 9) which agrees with the literature [22,44]. Figure 2B shows also that A39V-Cav1.2 also exhibits considerable C-lobe CDI (f_300_ = 0.28 ± 0.06, n = 9) which is not statistically different from WT-Cav1.2 channels. Overall, our data reveal a reduction in N-lobe CDI for A39V-Cav1.2, which implies a gain of function effect of this mutation. This is unexpected as Brugada syndrome is considered a loss-of-function disorder in the context of Cav1.2 channels [9,45].

**Figure 3:**
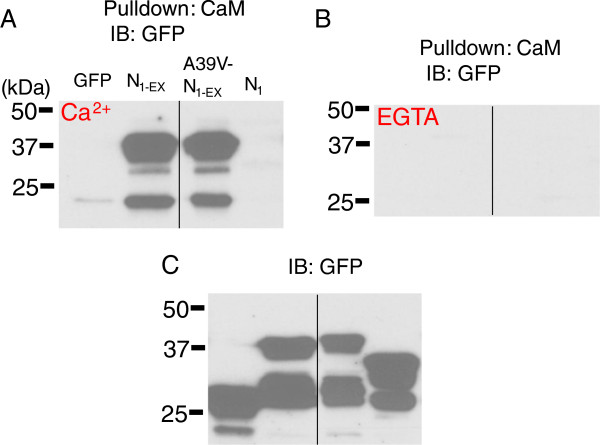
**The Brugada mutation A39V does not alter N-terminal binding to CaM. A)** CaM sepharose pull-down experiments of N_1-EX_, A39V-N_1-EX_ and N_1_ GFP fusion proteins in 0.5 mM Ca^2+^, or 5 mM EGTA **(B)** run on SDS-PAGE with corresponding lysates **(C)** and blotted for GFP. Black lines mark where the gel picture was cut and irrelevant samples removed. These experiments were performed twice each.

As N-lobe CDI was affected in the A39V mutant, we tested whether this was due to altered binding of CaM to the Cav1.2 N-terminus. We used CaM sepharose pulldowns to test whether CaM could differentially bind to fusion proteins of the distal N-terminus (methionine 1 to proline 101) of the channel. Figure 3A shows that both N_1-EX_ and A39V-N_1-EX_ GFP fusion proteins bound readily to CaM sepharose in 0.5 mM calcium. This binding was completely removed with 5 mM EGTA washes (Figure 3B). The smaller N_1_-GFP fusion protein (methonine 1 to lysine 63) did not bind CaM and agrees with our previous findings [23]. Altogether, these biochemical measurements show that A39V does not change the binding of CaM to the distal portion of the N-terminus. It is therefore unlikely that differential CaM binding explains the changes in N-lobe CDI observed for A39V-Cav1.2 channels.

### CaM mutants differentially affect the voltage-dependence and kinetics of activation for A39V and WT-Cav1.2 channels

**Figure 4:**
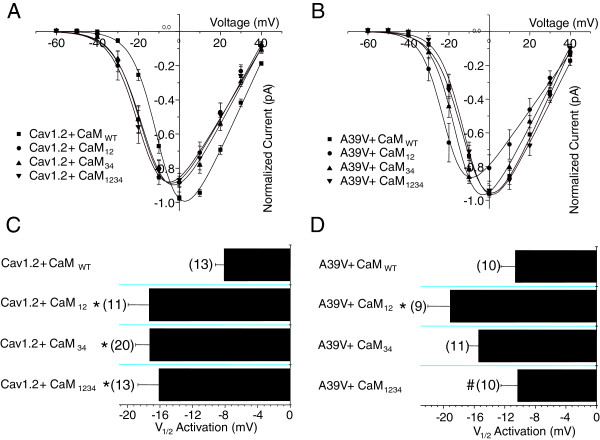
**CaM lobe mutants differentially shift the voltage dependence of activation for WT and A39V-Cav1.2 channels. A)** Current voltage relationships for WT-Cav1.2 channels expressed transiently in tsA-201 cells with Cavβ2a/Cavα2δ and recorded in barium with one of four CaM conditions: CaM_WT_, CaM_12_, CaM_34_, or CaM_1234_. All experiments were recorded with high calcium buffering intracellularly (10 mM BAPTA). **B)** Current–voltage relationships for A39V-Cav1.2 channels expressed as in **(A)** with one of four CaM conditions: CaM_WT_, CaM_12_, CaM_34_, or CaM_1234_. **C)** A bar graph displaying the half activation potentials for Cav1.2 channels recorded in barium. WT-Cav1.2 channels recorded with any CaM mutant have a significant leftward shift in the voltage-dependence of activation in barium compared to CaM_WT_ (*p ≤ 0.05 by one-way ANOVA). **D)** A bar graph displaying the voltage dependence of activation for A39V-Cav1.2 channels recorded in barium. A39V-Cav1.2 channels recorded with CaM_12_ have a significant leftward shift in the voltage-dependence of activation in barium compared to CaM_WT_ (*p ≤ 0.05 by one-way ANOVA), and CaM1234 (# p ≤ 0.05 by one-way ANOVA).

During the course of our experiments, we noticed that CaM lobe mutants affected the voltage-dependence of activation of Cav1.2 channels when bathed in extracellular barium solution. Figure 4A displays current–voltage (IV) relationships for WT-Cav1.2 channels expressed with Cavβ2a/Cavα2δ and either CaM_WT_, or one of CaM_12_, CaM_34_, or CaM_1234_. WT-Cav1.2 channels expressed with CaM_WT_ display a right-shifted IV relationship compared to all CaM mutants. Figure 4B displays the IV relationship for A39V-Cav1.2 under the same conditions, but in this instance, only the CaM_12_ condition shows an appreciable leftward shift relative to CaM_WT_. The bar graph in Figure 4C displays the Va for WT-Cav1.2 channels and reveals that all CaM mutants show a hyperpolarizing shift in the voltage-dependence of activation relative to the CaM_WT_ condition (p ≤ 0.05 by one-way ANOVA). For A39V-Cav1.2 only CaM_12_ shows a hyperpolarizing shift in Va compared to CaM_WT_ (p ≤ 0.05, one-way ANOVA) (Figure 4D). Altogether, these data indicate that alterations in CaM structure due to the functional elimination of either the N- or C-lobe EF hand motifs produce direct effects on Cav1.2 channel gating. These effects are present in the absence of calcium and can be modulated by the N-terminus of the channel as the A39V-Cav1.2 data indicate.

**Figure 5:**
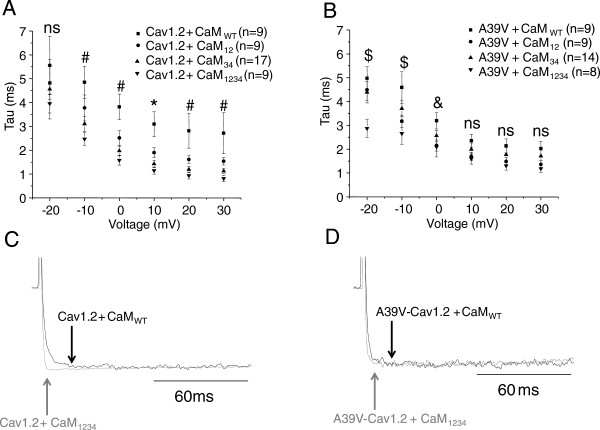
**Calmodulin lobe mutants differentially affect the kinetics of activation for WT and A39V-Cav1.2 channels in the absence of calcium. A)** Plot illustrating the time to maximum activation (Tau) at various voltages for WT-Cav1.2 channels expressed transiently in tsA-201 cells with Cavβ2a/Cavα2δ and recorded in barium with one of CaM_WT_, CaM_12_, CaM_34_ or CaM_1234_ and with 10 mM BAPTA intracellular. Voltages for which all mutant CaMs differ significantly from the CaM_WT_ condition (* p ≤ 0.05 by one-way ANOVA), and where CaM_34_ and CaM_1234_ differ from CaM_WT_ condition (# p ≤ 0.05 by one-way ANOVA). **B)** Plot for the kinetics of activation of A39V-Cav1.2 channels expressed as in **(A)**. Voltages for which CaM_1234_ differs significantly from the CaM_WT_ condition ($ p ≤ 0.05 by one-way ANOVA), and where CaM_12_ and CaM_1234_ differ from CaM_WT_ condition (& p ≤ 0.05 by one-way ANOVA). **C)** Sample traces of WT-Cav1.2 channels expressed with CaM_WT_ and CaM_1234_ at 10 mV. Note that the CaM_1234_ trace has been normalized to that of CaM_WT_ and that the arrows denote peak of activation for WT-Cav1.2 with either CaM_WT_ (black) or CaM_1234_ (grey). **D)** Sample traces of A39V-Cav1.2 channels expressed with CaM_WT_ and CaM_1234_ at 10 mV. Note that the CaM_1234_ trace has been normalized to that of CaM_WT_ and that the arrows denote peak of activation for A39V-Cav1.2 with either CaM_WT_ (black) or CaM_1234_ (grey).

We next examined whether the kinetics of Cav1.2 activation was affected by CaM mutants. Figure 5A shows that WT-Cav1.2 channels recorded with Cavβ2a/Cavα2δ and CaM_WT_ reach peak current amplitude much more slowly than all CaM mutants at 10 mV (p ≤ 0.05 by one-way ANOVA), and slower than CaM_34_ and CaM_1234_ at 0, 20 and 30 mV (p ≤ 0.05 by one-way ANOVA). These data indicate that mutating the C-lobe of CaM causes WT-Cav1.2 channels to open much quicker than they would otherwise at physiological depolarizations. Throughout our analysis we used a single exponential equation to fit the rapid rising phase of channel activation. In rare occasions, (1 cell out of 9 at +10 mV, for WT-Cav1.2), a second slower activation component was observed, but only in conditions with WT channels and CaM_WT_. We focused on our analysis only on the fast activation time constant.

As with the voltage-dependence of activation data, A39V-Cav1.2 channels behave differently from wild type channels with regards to their kinetics of activation in the presence of CaM mutants (Figure 5B). Specifically, at depolarized potentials A39V-Cav1.2 channels have similar kinetics of activation in the presence of wild type or mutant CaMs (p ≥ 0.05 by one-way ANOVA). This is also illustrated in the form of whole cell current traces depicted in Figures 5C and D at a test depolarization of +10 mV.

Altogether, our results reveal a previously unrecognized functional effect of CaM lobe mutants on Cav1.2 channel activation that can involve the N-terminus of the channel.

## Discussion

We have identified a novel effect of the pathophysiological mutation (A39V) through its reduction of N-lobe CDI of Cav1.2. Furthermore, our data reveal that mutant CaM molecules change activation gating for Cav1.2 channels even in the absence of calcium.

The observation that the A39V mutation reduced N-lobe CDI of Cav1.2 is surprising because Brugada syndrome is thought to involve a loss-of-function of these channels [9,45], rather than the gain of function observed here. It is important to note that the cDNA construct used in our studies corresponds to the neuronal form of the channel, and it is possible that the observed gain of function is specific to neuronal channels. Importantly, A39V is only thirteen residues away from a key amino acid residue that has been implicated in N-lobe CDI (W52). Indeed, Dick and colleagues [22] suggested that during N-lobe CDI, CaM leaves a C-terminal anchoring site upon calcium elevation to then interact directly with the N-terminal residue W52, which in turn promotes CDI. Our recent work has expanded this idea so that CaM binds W52 and a second more proximal residue C106, which then transduces the CDI signal into domain I of the channel, promoting closure. The observation that A39V does not affect CaM binding to the N-terminus of Cav1.2 (Figure 3) suggests that this residue may somehow be allosterically coupled to the CDI process. This could perhaps occur by partial immobilization of the N-terminus of Cav1.2, or by promoting additional intramolecular interactions within the N-terminus, or channel regions. For example, the N-terminus of Cav2.2 channels is capable of binding both the intracellular I-II linker and C-terminus [46]. As hydrophobic residues are often the anchor points for protein-protein interactions, it is possible that the A39V mutation may create a potential hydrophobic anchor.

How CaM molecules that are deficient in their ability to bind calcium affect Cav1.2 activation is particularly interesting. It is known that CaM_WT_ is capable of many conformations, most of which are calcium sensitive [29-32]. Conversely, CaM_1234_ (and potentially CaM_12_ and CaM_34_) display a different set of basic conformations [27,28] that may differ from calcium-free CaM_WT_. It is thus possible that the voltage-dependence and kinetics of Cav1.2 activation may be exquisitely sensitive to subtle changes in CaM structure. Figure 5 shows that the kinetics of Cav1.2 activation is altered by mutant CaMs. The immediacy of this kinetic change suggests that CaMs which participate in this process must be pre-bound to the channel. Because all of our experiments were performed in barium and with 10 mM BAPTA to buffer intracellular calcium, it is very unlikely that calcium has any role whatsoever in this effect. There is substantial evidence in the literature that in the absence of calcium CaM is tethered, or anchored to the C-terminus of VGCCs, specifically to the IQ domain [14,15] and upstream PCI region [24]. Cav1.2 channels also have an EF-hand motif in the proximal C-terminus which has been proposed to be involved in the transduction of CDI signals in the holo channel [39,47-49]. Immediately downstream of the EF-hand region is the PCI region which anchors the N-lobe of CaM in the absence of calcium [24]. The EF-hand of Cav1.2 has also been shown to modulate the voltage-dependence of activation with changing magnesium concentrations, a process which occurs also in the absence of calcium [50,51]. We propose that the inherent conformational differences of CaM mutants leverage the EF-hand region differently than CaM_WT_ and perhaps in a manner analogous to magnesium occupancy. The observation that this effect was abrogated in the A39V mutant may then indicate that this region may be functionally coupled to the C-terminus/CaM complex.

However, irrespective of the underlying molecular mechanisms, our data reveal that widely used CaM mutant constructs may exert effects on ion channel function that are independent of the inability of these proteins to bind calcium. This should be taken into consideration when interpreting data that rely on these CaM mutants.

## Conclusions

CaM lobe mutants are capable of altering both the voltage-dependent and kinetic properties of Cav1.2 channel activation in the absence of calcium. The Brugada syndrome mutation A39V reduces both N-lobe CDI and augments Cav1.2 channel activation.

## Methods

### cDNA constructs

Wild type (WT) rat calcium channel subunit cDNAs encoding Cav1.2, Cavβ2a and Cavα2δ1 subunits, as well as the pMT2 vector were donated by Dr. Terry Snutch (University of British Columbia, Vancouver, BC). Wild type CaM and a CaM mutant with four mutated EF hands (CaM_1234_) were a gift from Dr. John Adelman (Oregon Health Science University). GenBank™ accession numbers, or origins of the clones used are as follows: Cav1.2 [M67515], Cavβ2a [52], Cavα2δ1 [AF286488], and CaM [NP_114175.1]. Creation of A39V-Cav1.2 [38] as well as CaM_12_, CaM_34_ and the Cav1.2 N-terminal GFP fusion protein N_1_-GFP have been previously described [23]. N_1-EX_-GFP was generated by PCR off of WT-Cav1.2 channel cDNA and cloned into N1-GFP (Clontech) using BamHI/XhoI. Primers used to construct N_1-EX_-GFP were: ATATCTCGAGATGGTCAATGAAAACACG/TATAGGATCCCCGGGCGGCCGTGTGGCAGTTGTGC. All cDNAs were sequenced after cloning to verify fidelity.

### Tissue culture and transient transfection of tsA-201 cells

Human embryonic kidney tsA-201 cells were cultured and transiently transfected using the calcium phosphate method as described previously [53]. For immunoblotting 3ug of each cDNA was transfected per 10 cm plate. For electrophysiology experiments 6 ug of each alpha subunit and 3ug of Cavβ2a and Cavα2δ1 subunits were transfected per 10 cm plate. In addition, 125 ng of GFP was included in each electrophysiology transfection to identify transfected cells. For western blot experiments, cells were grown at 37°C for 48 h (75-85% confluence), while cells for electrophysiology were kept to low confluence and were grown for 72 hours at 28°C.

### Immunoblots and CaM pull-down assays

Cultured tsA-201 cells were transiently transfected as described above with cDNAs for immunoprecipitation/pull-down assays and were lysed with a modified RIPA buffer (in mM; 50 Tris, 130 NaCl, 0.2% triton X-100, 0.2% NP-40, 5 EGTA, or 0.5 Ca^2+^, pH 7.4). Lysis was carried out on ice for 15 min after which cells were centrifuged at 13,000 rpm for 5 min at 4°C. Supernatants were transferred to new tubes and solubilized proteins were mixed with CaM Sepharose 4B beads (GE Healthcare Life Sciences) for pull-down assays overnight while tumbling at 4°C. Pulldowns were washed three times with either 0.5 mM Ca^2+^, or 5 mM EGTA lysis buffer, eluted with 2X Laemmli sample buffer and incubated at 96°C for 10 min. Eluted samples were loaded on the appropriate percentage Tris-glycine gel and resolved using SDS-PAGE. Samples were transferred to 0.45 μm PDVF membranes (Millipore) and immunoblot performed using 1/1000 anti-GFP (Santa-Cruz-8334). GE-Healthcare horseradish peroxidase-linked secondary antibodies (rabbit) was used at 1/5000 dilution. Total inputs were taken from whole cell samples and represented 2.5% of total protein.

### Voltage-clamp recordings

Glass cover slips carrying cells WT or mutant Cav1.2 channels were transferred to a 1.5 ml recording chamber and external recording solution consisting of 20 mM BaCl_2_ or 20 mM CaCl_2_, 1 mM MgCl_2_, 10 mM HEPES, 10 mM Glucose and 136 mM CsCl (pH 7.4 adjusted with CsOH) was perfused. Micro-electrode patch pipettes were pulled and polished using a DMZ- Universal Puller (Zeitz Instruments GmbH) to a typical resistance of 3–5 Ώ. Low calcium buffering internal pipette solution consisted of 141 mM CsCH_3_SO_3_, 0.5 mM EGTA, 4 mM MgCl_2_ and 10 mM HEPES (pH 7.2 adjusted with CsOH). High calcium buffering internal solution was prepared in the same way however less CsCH_3_SO_3_ (131 mM) was used to offset the increase in calcium buffer concentration of 10 mM BAPTA. Added daily to internal solution was 5 mM Di-Tris-Creatine Phosphate, 2 mM Tris-ATP and 0.5 mM Na-GTP.

Whole cell patch clamp recordings were performed in voltage-clamp mode using an Axopatch 200B amplifier (Axon Instruments) linked to a personal computer with pCLAMP software version 9.2. Series resistance was compensated by 85%, leak currents were negligible, and the data were filtered at 5 kHz. Individual GFP expressing cells were held at −100 mV and pulsed in 10 mV increments from −60 to +60 mV, for a period of 1 second. Individual pulses were separated by 15 s to enable full channel recovery. Only those cells whose whole cell current voltage relationships could be fit with the modified Boltzmann equation, I = (1/(1 + exp^(−(Va-V)/S))^)*(V-E_rev_)*G_max_, where ‘I’ is current, ‘V_a_’ is half-activation potential, ‘V’ is membrane potential, ‘E_rev_’ is reversal potential, S is the slope factor, and ‘G_max_’ is slope conductance, were used for determination of voltage-dependent properties. IV curves displayed in Figure 4 are ensemble fits, and because of variance in the data, not all conditions plotted reach a normalized value of −1. Determination of Va was always determined by fitting individual whole cell current–voltage relationships, rather than using the ensemble fits.

For CDI experiments only cells with > 80pA of Ba^2+^ current proceeded to recordings in Ca^2+^. In order to quantify CDI we used a previously described method of paired analysis [21]. In this method the fraction of current remaining at 300 ms (r300) in Ca^2+^ is subtracted from the current fraction remaining at 300 ms in Ba^2+^. The difference obtained between the two charge carriers represents additional inactivation promoted by Ca^2+^ (f_300_), or rather CDI. Because Ca^2+^ conductance in the solutions used was maximal at 10 mV, the −100 to 10 mV (1 sec) pulse was used for determining degree of CDI.

### Data analysis

All electrophysiological data were analyzed using Clampfit version 10.2 (Axon Instruments) and plotted in Origin 9 (Origin Lab Corporation). Statistical analyses for both biochemical and electrophysiological data were carried out using Origin 9. All sample means are reported as +/−SEM. Statistically significant differences between means were assessed using student’s *t*-test, or one-way ANOVA at the 95% confidence level (followed by Tukey’s test), as appropriate.

### Ethical standards

All experiments performed in this manuscript comply with the laws of Canada.

## Abbreviations

CaM: Calmodulin; CDF: Calcium dependent facilitation; CDI: Calcium dependent inactivation; VGCC: Voltage gated calcium channel; Ca2+/CaM: Calcium/calmodulin; VDA: Voltage dependent activation; WT-Cav1.2: Wild type Cav1.2; VDI: Voltage dependent inactivation; IV: Current voltage; Va: Half activation potential.

## Competing interests

The authors declare that they have no competing interests.

## Authors’ contributions

All authors were involved in the design of the study. BAS designed and carried out electrophysiology experiments, and drafted the manuscript. IAS designed and conducted biochemistry experiments. GWZ directed the study and edited the manuscript. All authors read and approved the final manuscript.
